# Age-Dependent Effects of Haptoglobin Deletion in Neurobehavioral and Anatomical Outcomes Following Traumatic Brain Injury

**DOI:** 10.3389/fmolb.2016.00034

**Published:** 2016-07-19

**Authors:** Alexander V. Glushakov, Rodrigo A. Arias, Emanuela Tolosano, Sylvain Doré

**Affiliations:** ^1^Department of Anesthesiology, Center for Translational Research in Neurodegenerative Disease, University of Florida College of MedicineGainesville, FL, USA; ^2^Departments of Molecular Biotechnology and Health Sciences, University of TorinoTorino, Italy; ^3^Departments of Anesthesiology, Neurology, Psychiatry, Psychology, Pharmaceutics and Neuroscience, University of Florida College of MedicineGainesville, FL, USA

**Keywords:** controlled cortical impact, Iba1, GFAP, gliosis, hemoglobin, hemorrhage, trauma

## Abstract

Cerebral hemorrhages are common features of traumatic brain injury (TBI) and their presence is associated with chronic disabilities. Recent clinical and experimental evidence suggests that haptoglobin (Hp), an endogenous hemoglobin-binding protein most abundant in blood plasma, is involved in the intrinsic molecular defensive mechanism, though its role in TBI is poorly understood. The aim of this study was to investigate the effects of Hp deletion on the anatomical and behavioral outcomes in the controlled cortical impact model using wildtype (WT) C57BL/6 mice and genetically modified mice lacking the Hp gene (Hp^−∕−^) in two age cohorts [2–4 mo-old (young adult) and 7–8 mo-old (older adult)]. The data obtained suggest age-dependent significant effects on behavioral and anatomical TBI outcomes and recovery from injury. Moreover, in the adult cohort, neurological deficits in Hp^−∕−^ mice at 24 h were significantly improved compared to WT, whereas there were no significant differences in brain pathology between these genotypes. In contrast, in the older adult cohort, Hp^−∕−^ mice had significantly larger lesion volumes compared to WT, but neurological deficits were not significantly different. Immunohistochemistry for ionized calcium-binding adapter molecule 1 (Iba1) and glial fibrillary acidic protein (GFAP) revealed significant differences in microglial and astrocytic reactivity between Hp^−∕−^ and WT in selected brain regions of the adult but not the older adult-aged cohort. In conclusion, the data obtained in the study provide clarification on the age-dependent aspects of the intrinsic defensive mechanisms involving Hp that might be involved in complex pathways differentially affecting acute brain trauma outcomes.

## Introduction

Traumatic brain injury (TBI) is one of the major causes of morbidity and mortality in the United States and worldwide with no current effective treatment. It is widely recognized that chronic neurological and psychological disabilities following TBI largely result from potentially preventable or treatable secondary pathophysiological cascades, so-called secondary injuries, initiated by the acute brain trauma. However, despite extensive research and promising results obtained in preclinical studies, numerous clinical trials failed primarily due to the heterogeneity and complexity of the TBI and mechanisms underlying acute vs. chronic anatomical pathologies and neurological deficits. Secondary injuries resulting from acute TBI, such as mechanical brain damage and intracranial hemorrhages, are triggered by complex coexisting pathways involving excitotoxicity, oxidative damage, and neuroinflammatory cascades (McIntosh et al., 1996; Diaz-Arrastia et al., 2000; Saatman et al., 2008). On the other hand, the presence of acute subarachnoid or, to a lesser degree, parenchymal hemorrhages following TBI is highly associated with brain-injury severity and chronic cognitive and physical disabilities; and it is believed that the mechanisms are mainly initiated by the toxicity of hemoglobin and its heme-containing breakdown products released into the brain tissues from erythrocytes during hemolysis of cerebral hematomas (Xi et al., 1998; Bhasin et al., 2002), causing oxidative damage to macromolecules such as lipids, proteins, and nucleic acids (Nakamura et al., 2005, 2006), as well as activation caspase proteolysis (Regan and Panter, 1993; Wang et al., 2002), resulting in disruption of the blood-brain barrier (Xi et al., 1998; Bhasin et al., 2002; Keep et al., 2008) and neuronal death (Koeppen et al., 1995; Aronowski and Hall, 2005; Xi et al., 2006).

One of the critical mechanisms involved in deactivation of cell-free hemoglobin in the mammalian body during the hemorrhagic event is formation of highly stable complexes of hemoglobin with haptoglobin (Hp)—an endogenous hemoglobin-binding protein present in blood plasma and almost absent within the brain itself (Wada et al., 1970; Philippidis et al., 2004; Schaer et al., 2006)—and subsequent clearance of the Hp-hemoglobin complexes primarily by tissue macrophages and circulating monocytes, and likely by other cell types such as astrocytes and microglial cells that are mediated via CD163 (Liu and Sturner, 1988; D'Armiento et al., 1997; Ascenzi et al., 2005; Schaer et al., 2005, 2007; Zhang et al., 2012). However, at this point, the contribution of Hp to acute brain injury is still not fully understood (D'Armiento et al., 1997).

The changes in Hp expression have been shown in various disorders and diseases associated with inflammation (Carter and Worwood, 2007). It is well recognized that under physiological conditions the plasma pool of Hp is relatively high and its levels further increase up to 10-fold in different types of injuries associated with hemorrhages as a part of the acute phase-2 response (Petersen et al., 2004). Early studies have shown that serum Hp concentrations are increased in patients with severe head injuries and that the serum Hp level could potentially be a predictive biomarker of the hemorrhagic brain-lesion severity (Auer and Petek, 1978). In both physiological and pathophysiological conditions, Hp is synthesized mainly by hepatocytes and then released to the peripheral circulation (Bowman and Kurosky, 1982; Hoj et al., 1984; Yang et al., 2013). However, some studies suggest that Hp may have very limited expression within brain-cell types. The Hp mRNAs were found in the human retinal pigment epithelial cell line and post-mortem neural retina (Chen et al., 1998). Expression of Hp has also been demonstrated in human glioblastoma tissue but not in normal brain samples (Kumar et al., 2010). Few groups have documented that Hp is present within the brain of rodents following brain injuries (Lee et al., 2002; Zhao et al., 2009, 2011). Mouse studies from Dr. J. Aronowski and colleagues suggested that Hp is expressed and secreted by brain oligodendrocytes after experimental intracerebral hemorrhage and that brain-derived Hp plays a significant role in the protection of brain cells after injury (Zhao et al., 2009, 2011) in addition, a rat study by Dr. Kim and colleagues suggested Hp expression in the hippocampus following brain ischemia (Lee et al., 2002). Increased Hp immunoreactivity and upregulation of Hp mRNA in reactive astrocytes have been shown in an experimental ischemia model, suggesting *de novo* Hp synthesis in the brain (Lee et al., 2002). On the other hand, a human study of subarachnoid hemorrhage suggested an influx of Hp from peripheral circulation into cerebrospinal fluid, and that the intrathecal Hp-scavenging system could have limited capacity (Galea et al., 2012). Previously, Hp levels in cerebrospinal fluid have been proposed as a biomarker of blood-brain barrier disruption (Chamoun et al., 2001). It has also been demonstrated that, following TBI, the increase in brain Hp levels occurs due to extravasation of Hp, as well as other types of plasma proteins, into brain parenchyma resulting from the blood-brain barrier breakdown associated with intracranial hemorrhage and subsequent uptake of the plasma proteins by reactive astrocytes (Liu and Sturner, 1988).

Current interest is driven by recent studies suggesting that Hp phenotypes, primarily associated with different affinities to bind free hemoglobin and affinity of the hemoglobin-Hp complex to its receptors, may be associated with differential outcomes in subarachnoid hemorrhages (Chaichana et al., 2007, 2010; Leclerc et al., 2015) and that Hp may play an important role in the development of secondary injuries, particularly delayed arterial vasospasm and brain ischemia (Nonaka et al., 1979; Borsody et al., 2006). Of interest, post-traumatic cerebral vasospasm is a common complication of TBI, with incidences ranging from 2 to 63% mainly depending on the severity of injury and the method of diagnostics (Macpherson and Graham, 1978; Taneda et al., 1996; Mattioli et al., 2003). Although there is a strong association of cerebral vasospasm with traumatic subarachnoid hemorrhage (Macpherson and Graham, 1978; Gomez et al., 1991; Steiger et al., 1994; Kordestani et al., 1997; Aminmansour et al., 2009), it is also common in patients with subdural hematomas, intraventricular hemorrhage, and contusions (Mattioli et al., 2003; Oertel et al., 2005; Kalanuria et al., 2013). In general, subarachnoid hemorrhages are associated with extremely high rates of mortality of about 45–50%, significant morbidity exists among survivors (van Gijn et al., 2007; Bederson et al., 2009) and chronic disabilities are common (Kantor et al., 2014). It should be noted that subarachnoid hemorrhage associated with TBI accounts for about half of all cases, and subsequent cerebral vasospasm following both aneurysmal and traumatic subarachnoid hemorrhages is among the leading causes of morbidity and mortality with no proven effective treatment (Suarez et al., 2006; Amyot et al., 2015). Thus, identifying the mechanisms underlying TBI pathologies and intrinsic, potentially protective responses involving acute reactants, such as Hp (Vejda et al., 2002; Campbell et al., 2005), will provide new insights into the development of novel strategies for TBI treatment.

Experimental and clinical data suggest that Hp phenotypes are differentially associated with occurrence of cerebral vasospasm, a common complication of subarachnoid hemorrhage, and that the patients with increased risk might be identified based on their Hp genotype (Chaichana et al., 2007, 2010; Leclerc et al., 2015). The human and mouse Hp cDNAs share homology of >80%, and although mice are homomorphic for the Hp genotype expressing only a “high” affinity Hp1–1 phenotype, Hp phenotypes were associated with differential neuropsychological outcomes after TBI; however, in this case, the Hp 1–1 phenotype, which is characterized by the highest affinity to hemoglobin, was associated with worse outcomes (Lee et al., 2002; Anderson et al., 2009).

Although Hp is implicated in the pathophysiology of different brain injuries, the published data suggest that its roles are complex, that upregulation of Hp might have either or both beneficial and detrimental effects, and that the outcomes might be predisposed by certain Hp genotypes. Thus, taking into account the heterogeneity of Hp phenotypes in humans, the goal of this preclinical study was to investigate the role of the “high” efficacy Hp phenotype in a controlled cortical impact (CCI) model of TBI using wild type (WT) C57BL/6 mice and genetically modified mice of the same background lacking the Hp gene (Hp^−∕−^) and comparing anatomical and gliosis outcomes in two different adult-age cohorts.

## Materials and methods

### Experimental animals

Two matched age cohorts [i.e., 2–4 mo-old (adult) and 7–8 mo-old (older adult)] of WT and Hp^−∕−^ C57BL/6 male mice were used in the study. Hp^−∕−^ mice were maintained in the in-house facility from the breeding stock provided by Dr. E. Tolosano. The number of WT animals per group used for assessment of anatomical and immunohistochemical analyses were as follows: sham *n* = 5 and *n* = 5, and CCI *n* = 8 and *n* = 10 in the adult (2–4 mo-old) and older adult (7–8 mo-old) age cohorts, respectively. The number of Hp^−∕−^ mice per CCI group used for assessment of anatomical and immunohistochemical outcomes per group for analyses were *n* = 9 and *n* = 4 in the adult and older adult age cohorts, respectively. In addition, small groups of sham Hp^−∕−^ mice of both ages were used to confirm negligible effects of craniotomy. For assessment of the behavioral outcomes, the mice numbers were slightly inflated due to intrinsic mouse variability of the behavioral outcome measures and confirmation of behavioral test results. The numbers of animals used in the analyses are indicated in the figure legends. The experiments and procedures were carried out in strict accordance with the recommendations in the Guide for the Care and Use of Laboratory Animals of the National Institutes of Health. All procedures used in this study were approved by The University of Florida Institutional Animal Care and Use Committee. All surgery was performed under anesthesia, and all efforts were made to minimize the pain and distress of the experimental animals.

### CCI procedures

In this study, we used the same CCI or sham procedures as previously described (Glushakov et al., 2013). Briefly, mice were anesthetized with 4% isoflurane and maintained with 2% isoflurane during all surgery procedures. Mice were placed in the stereotaxic apparatus and the experimental contusive TBI of mild-to-moderate severity was induced using a conventional CCI model (PCI3000 PinPoint Precision Cortical Impactor, Hatteras Instruments, Cary, NC, USA) with an impact tip diameter of 3 mm, velocity of 3 m/s, and compression distance and time of 1 mm and 100 ms, respectively (Yu et al., 2009). The experimental injury in all cases was induced in the right hemisphere. Sham-injured mice underwent only the anesthesia and craniotomy surgeries. After closing the incision, the mice were removed from the stereotaxic apparatus, received an intraperitoneal injection of warm saline to prevent dehydration, and were placed into a temperature-controlled recovery chamber for at least 1 h before being transferred to the animal housing facility.

### Neurobehavioral deficits

Neurobehavioral deficits were assessed 24 and 48 h after CCI or sham procedures using a 24-point Neurological Deficit Score (NDS) scale as described in detail elsewhere (Glushakov et al., 2013). Briefly, the assessment comprised six tests, including body symmetry, gait, circling behavior, climbing on the incline plane, and tail suspension tests to access compulsory circling and front limb symmetry. Each of these individual tests was scored between 0 and 4 points for normal performance (score 0) and according to criteria of gradually increased severity from score 1 to 4; the NDS was calculated as a sum score obtained from the assessment. Prior to behavioral testing, the animals were allowed to acclimate in the testing room for about 30 min. For quantitative assessment of stereotypic activity and circling behavior, the neurobehavioral assessment was based on the moving pattern criteria used in the individual circling behavior test performed on the open bench top, which included a part of the NDS test. The circling behavior test preceded all other behavioral tests. The mouse was placed on the elevated open rectangular plane surface and allowed to move freely for at least for 2–5 min depending on the animal's moving activity. The activity was videotaped from above and analyzed off-line by a blinded examiner. The number of left and right turns was counted for at least for 2 min or for a longer time period to obtain a total number of turns of at least about 20. Preferential turns to one side are indicative of stereotypic movement or circling behavior. The activity values were calculated as a total number of left and right turns per minute, and the circling behavior values were calculated as a percentage of right turns.

### Brain histopathology and immunohistochemistry

All mice used in the study were euthanized at 48 h and processed for quantitative stereological brain pathology using cresyl violet staining and immunohistochemistry as described before (Glushakov et al., 2014). All slides (eight 30-μm-thick brain sections per slide cut from the same animal and spaced about 500 μm apart) were scanned using ScanScope (Aperio Technologies, Vista, CA, USA) and analyzed using ImageScope software (Aperio) in a blinded manner. The volumes of cortical lesions and tissue loss were estimated within 2-mm-thick brain segments positioned between the bregma and 2 mm posterior to the bregma (i.e., bregma coordinates from 0 to −2 mm). Cortical lesions were defined as histological alterations in the ipsilateral cortical structures within the site of impact and proximate areas characterized by abnormal cellular morphology and irregular cell density compared to the histological characteristic of their corresponding contralateral counterparts, and, in some cases, the presence of diffuse parenchymal hemorrhages and small hematomas, whereas cavitation was defined as complete loss of cortical tissue or the presence of enlarged hematomas without evident existence of cresyl violet staining. The volume of cortical injury was defined as a combined volume comprising the volumes of cortical lesions and cavitation. Hippocampal edema was assessed by measuring the volumes of ipsilateral and contralateral hippocampi as described before (Glushakov et al., 2013).

Reactive astrogliosis and microglial activation were assessed using immunohistochemistry for glial fibrillary acidic protein (anti-GFAP, 1:1000 DAKO, Carpinteria, CA, USA) and for ionized calcium-binding adapter molecule 1 (anti-Iba1, 1;100, Wako Bioproducts, Richmond, VA, USA), respectively; vector kits were then used for DAB antigen visualization (Vector Laboratories, Burlingame, CA, USA). Immunoreactivity was calculated as a sum of positive and strong positive pixels in rectangular selections (500 × 500 μm) in selected brain regions with characteristic microglial and astrocytes morphological changes. The brain regions were identified using Allen Mouse Brain Atlas. To compare the astroglial and microglial reactivity distribution in the anteroposterior directions from the midline of cortical impact (between −1 and −2 mm from the bregma) coordinates, the assessment was performed in about 1-mm-thick brain segments starting from the bregma posteriorly (from 0 to −4 bregma coordinates). To calculate relative immunoreactivity, the images of two to three sections for each segment from each brain slide (i.e., up to eight sections per animal) were analyzed separately and the mean data for each section in each animal were included in group analysis.

### Statistical analyses

The power analysis to determine approximate minimal group size was performed based on the assumption of obtaining statistical significance of anticipated differences of means equal to at least 1.5 standard deviation with a significance level of α = 0.05 and a power of 1−ß = 0.80. To compare the differences between anatomical and immunohistochemical outcomes and locomotor activity in mice groups with different surgical procedure (i.e., sham and CCI), genotype (i.e., WT and HP^−∕−^), and age (i.e., young adult and older adult), the data were analyzed using multi-factor analysis of variance (ANOVA) performed by mixed model regression data analysis and Turkeys *post-hoc* comparison test between matched groups. To compare the differences between non-parametric NDS data, a series of ANOVA on ranks and Dunn's *post-hoc* test were been performed, taking into consideration multiple aforementioned factors (i.e., surgical procedure, age, and genotype) and repeated measurements. All parametric data are presented as the mean ± standard error, and non-parametric data as median, range, and interquartile range (IQR). *P*-values less than 0.05 were considered significant (Macleod et al., 2009). Statistical analyses were performed using JMP (SAS Institute Inc., Cary, NC, USA) and GraphPad (GraphPad Software Inc., La Jolla, CA, USA) software.

## Results

### Effects of the Hp knockout on neurobehavioral outcomes following CCI in different age cohorts

**Figure 1:**
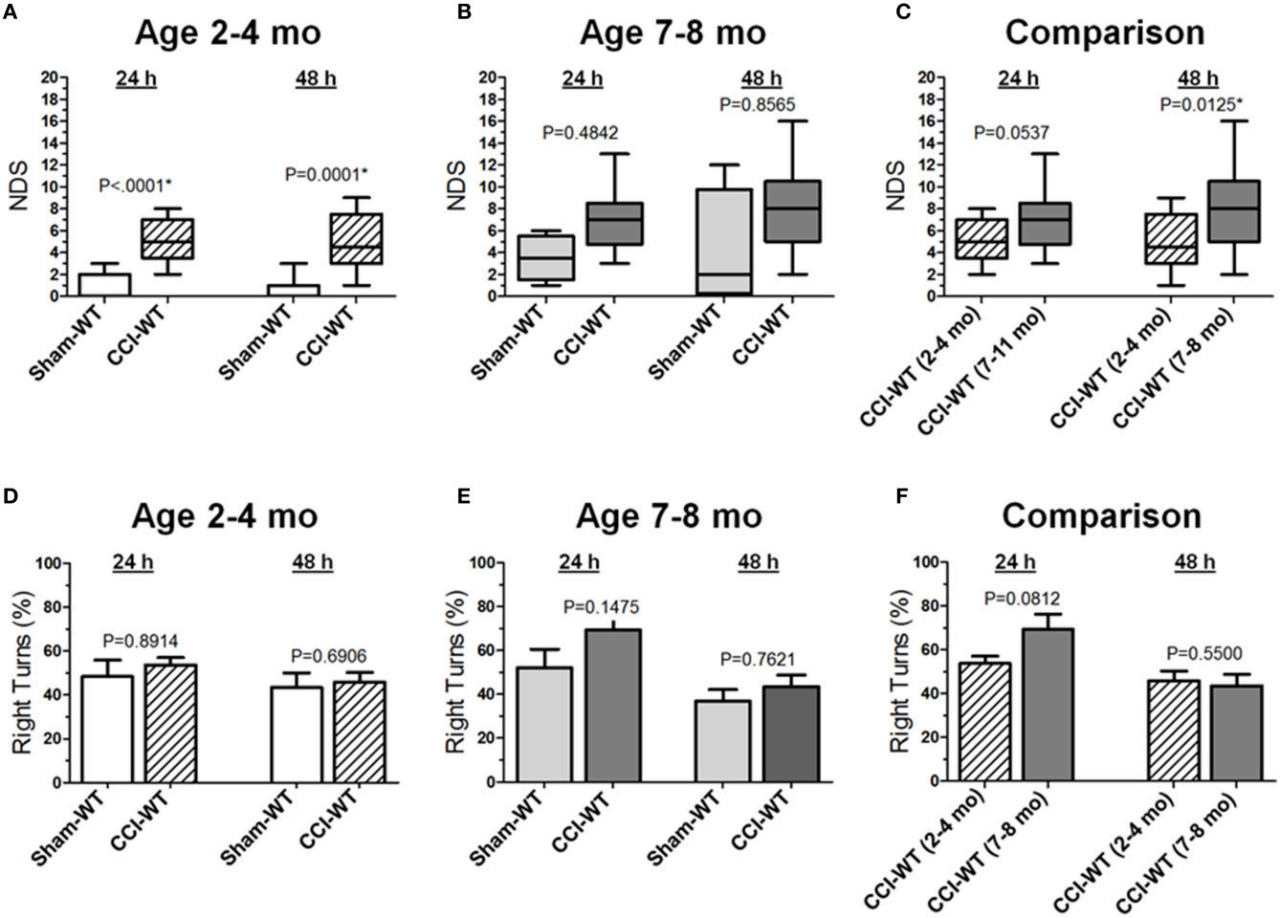
**Age-dependent effects of the experimental TBI on neurobehavioral outcomes**. **(A-C)** Comparison of NDS assessed at 24 and 48 h after the experimental injury between sham- and CCI-injured WT mice in adult and older adult age cohorts **(A,B)**, and between CCI-injured WT mice of different ages **(C)**. Statistical comparison of combined data presented in the **(A-C)** using a series of non-parametric multi-factor ANOVA with repeated measurement structure. The numbers shown on the graphs represent *P*-values from the *post-hoc* statistical analyses performed using non-parametric Dunn's rank sum test. **(D-F)** Comparison of the circling behavior presented as the fraction of the right turns between sham- and CCI-injured WT mice in adult and older adult age cohorts **(D,E)**, and between CCI-injured WT mice of different ages **(F)**, respectively. Statistical comparison of combined data using mixed model multi-factor ANOVA with repeated measurement structure. *P*-values shown on the graphs between two groups used for pairwise comparison were obtained from the statistical analyses using the *post-hoc* Student's *t*-test. In all panels, the numbers of animals per sham and CCI experimental groups of WT mice used for analyses were as follows: *n* = 6 and *n* = 18 in the adult mice age cohort (2–4 mo-old), and *n* = 5 and *n* = 13 in the older adult mice age cohort (7–8 mo-old), respectively.

Neurological deficits after experimental TBI were assessed 24 and 48 h after CCI using the NDS and the differences between multiple group factors, including surgery type and age with repeated outcome measures, which were assessed using a series non-parametric multi-factor ANOVA on ranks and *post-hoc* Dunn's rank sum test. In adult mice (2–4 mo-old), the median NDS values in the CCI group at 24 and 48 h were 5 at both time points (IQRs: 4–7 and 3–8, respectively), and these values were significantly different (*P* < 0.0001 and *P* = 0.0001) from those observed in the mice from the sham group with median values of 0 at both time points (IQRs: 0–2 and 0–1, respectively; Figure 1A). In older adult mice (7–8 mo-old), the median NDS values at 24 and 48 h were 7 (IQR: 5–8) and 8 (IQR: 5–10), respectively, although the NDS values in the CCI group were not significantly different from those observed in the sham group (Figure 1B). Although the median values were similar at both time points (7 and 8), the NDS 48 h after CCI was characterized by increased variability (IQRs: 2–5 and 0–10 24 and 48 h after surgery, respectively). In this age cohort, the same animals 48 h after surgery showed worse NDS in the CCI and sham groups, resulting in a wider NDS value range and a lack of statistical significance. As compared to the 48 h NDS scores, the median values were similar to those at the 24 h time point. In addition, further analyses of time dependency based on 24 and 48 h comparisons revealed no significant differences between NDS values at these time points in the sham or CCI group of the adult or older adult mice cohort (Figure 1C). The NDS values for the sham and CCI groups were higher compared to their counterpart groups of 2–4 mo-old mice. These values were significantly different (*P* < 0.05) only at the 48 h time point. No significant differences were observed between the sham groups of adult and older adult animals at any time point.

Based on the anatomical brain pathology of marked lesions and tissue loss in the ipsilateral motor cortex observed in the CCI model, we performed an analysis of locomotor activity pattern by counting the number of left and right turns of free-moving mice to quantitatively assess the circling behavior associated with unilateral impairment of this brain region (Figures 1D,E). The analyses of stereotypic movement behavior and activity in the group of mice used for NDS assessment using mixed model multi-factor ANOVA revealed a significant temporal effect (*P* = 0.0009), although the *post-hoc* comparison between matching groups using Student's *t*-test revealed no significant differences between groups. There were no significant differences between proportions of left and right turns between the sham and CCI groups 48 h after experimental injury or in each group between the 24 and 48 h time points in both age cohorts. Similarly, in both age cohorts, there were no significant differences between locomotor activity calculated as the number of left and right turns or total number of left and right turns combined between the sham and CCI groups or in each group between 24 and 48 h time points (data not shown). To compare the differences between experimental groups, the values of moving symmetry were expressed as a percentage fraction of right turns. Mixed model multi-factor ANOVA revealed a significant temporal effect (*P* = 0.0009). However, *post-hoc* comparison between matching groups also revealed no statistically significant differences between outcomes in the CCI groups from adult and older adult cohorts at both 24 and 48 h time points (Figure 1F).

**Figure 2:**
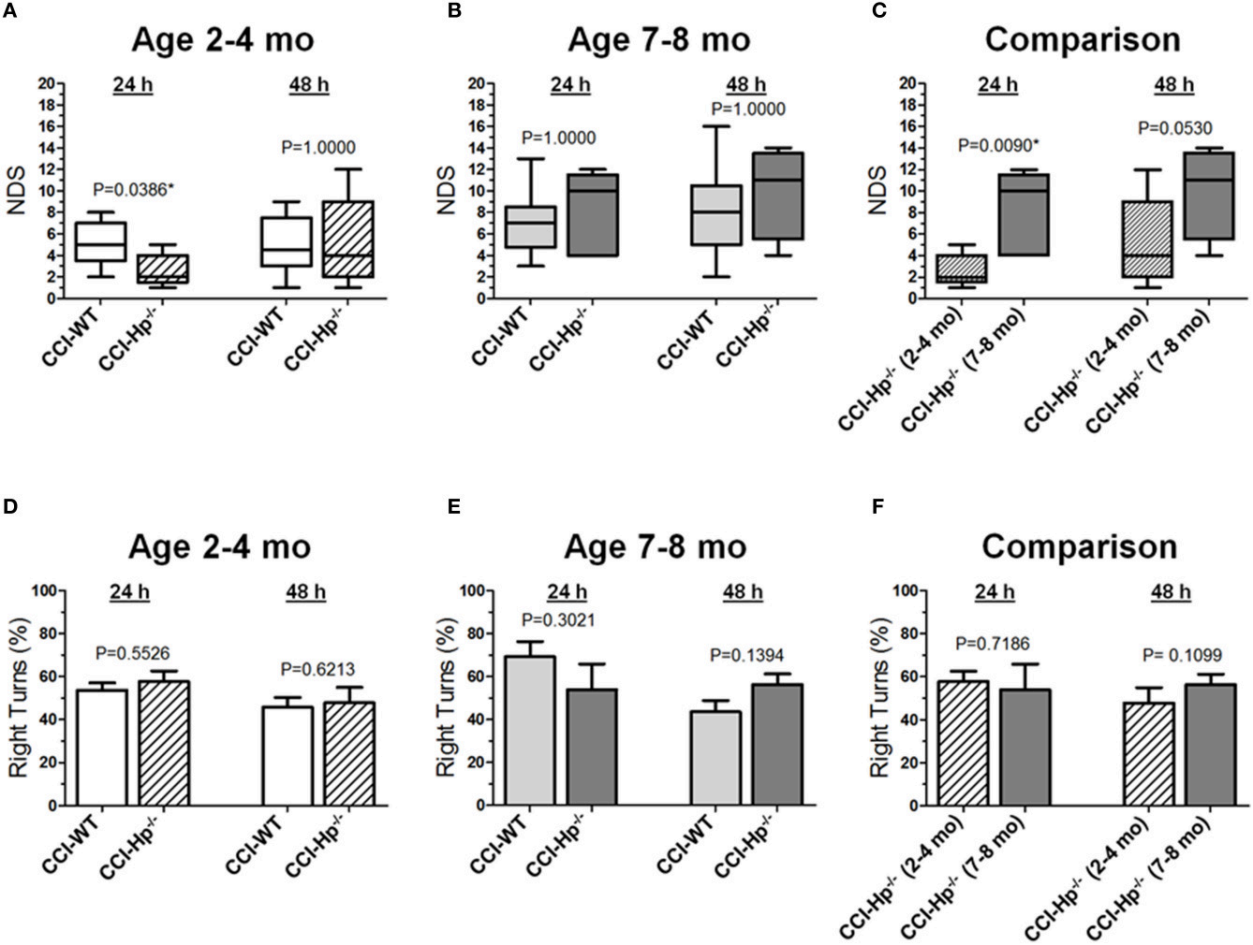
**Effects of the Hp knockout on neurobehavioral outcomes following CCI in different age cohorts**. **(A-C)** Comparison of NDS assessed at 24 and 48 h after the experimental injury between CCI-injured WT and HP^−∕−^ mice in adult and older adult age cohorts **(A,B)**, and between CCI-injured HP^−∕−^ mice of different ages **(C)** Statistical comparison of combined data presented in the **(A-C)** using a series of non-parametric multi-factor ANOVA with repeated measurement structure. The numbers shown on the graphs represent *P*-values from the *post-hoc* statistical analyses performed using non-parametric Dunn's rank sum test. **(D-F)** Comparison of the circling behavior presented as the fraction of the right turns between CCI-injured WT and Hp^−∕−^ mice in adult and older adult age cohorts **(D,E)**, and between CCI-injured WT mice of different ages **(F)**, respectively. Statistical comparison of combined data using mixed model multi-factor ANOVA with repeated measurement structure. *P*-values shown on the graphs between two groups used for pairwise comparison were obtained from the statistical analyses using the *post-hoc* Student's *t*-test. In all panels, the numbers of animals per CCI experimental groups of WT and Hp^−∕−^ mice used for analyses were as follows: *n* = 6 and *n* = 18 in the adult mice age cohort (2–4 mo-old), and *n* = 16 and *n* = 13 in the adult mice age cohort (2–4 mo-old), and *n* = 13 and *n* = 5 in the older adult mice age cohort (7–8 mo-old), respectively.

Figures 2A-C demonstrate the comparison of NDS between WT and Hp^−∕−^ CCI-injured mice. A series of non-parametric multi-factor ANOVA and *post-hoc* comparison between matching groups using Dunn's *t*-test revealed significant differences in NDS of the Hp^−∕−^ mice from the CCI group in the adult age cohort (*P* = 0.0386), whereas 48 h after injury, the differences between WT and Hp^−∕−^ were no longer significant, suggesting possible neurological deterioration. Significantly lower values in the Hp^−∕−^ group in the adult mice cohort suggest an improved neurological outcome 24 h after experimental injury, whereas no significant differences in the NDS were observed between WT and HP^−∕−^ CCI-injured mice in the older adult cohort at the 24 and 48 h time points (Figure 2B). There was a significant difference between the NDS in the CCI-injured mice from the adult and older adult cohorts 24 h after injury (*P* = 0.0094), whereas at 48 h, the increase in the NDS was also no longer significant (Figure 2C). In addition, to determine possible effects of surgery alone on the neurobehavioral outcomes in Hp^−∕−^ mice at the two ages, the NDS test was performed in small groups of animals that underwent sham surgery (*n* = 4 in adult and *n* = 2 in older adult cohorts). The data revealed no substantial increases in NDS scores in sham Hp^−∕−^ mice, which ranged from 0 to 1 in adult and from 0 to 4 in the older adult cohort at 24 and 48 h time points (data not shown). No circling behavior was detected in any Hp^−∕−^ group after CCI, and no significant differences were observed between WT and Hp^−∕−^ mice in both age cohorts (Figures 2D,E) or between different age cohorts of Hp^−∕−^ mice (Figure 2F), although a significant temporal effect (*p* = 0.0009) on the moving symmetry was revealed by mixed model multi-factor ANOVA.

### Effects of the Hp knockout on anatomical outcomes following CCI in the different age cohorts

In the first set of experiments, we performed tests to determine differences in anatomical deficits after CCI in 2–4 (adult) and 7–8-mo-old (older adult) WT mice; subsequent comparisons were then made between WT and Hp^−∕−^ mice in each age cohort. Macroscopically, the overall brain pathology from CCI with mild-to-moderate TBI parameters 48 h post-injury was characterized by neuronal death, loss of cortical tissue, and, to a lesser degree, partial loss of hippocampal tissue or hippocampal distortion with altered structural tissue integrity as compared to the contralateral hippocampus or hippocampi of animals from the sham-injury group. These anatomical brain changes are consistent with our previous published data using the same CCI parameters (Glushakov et al., 2013, 2014, 2015).

**Figure 3:**
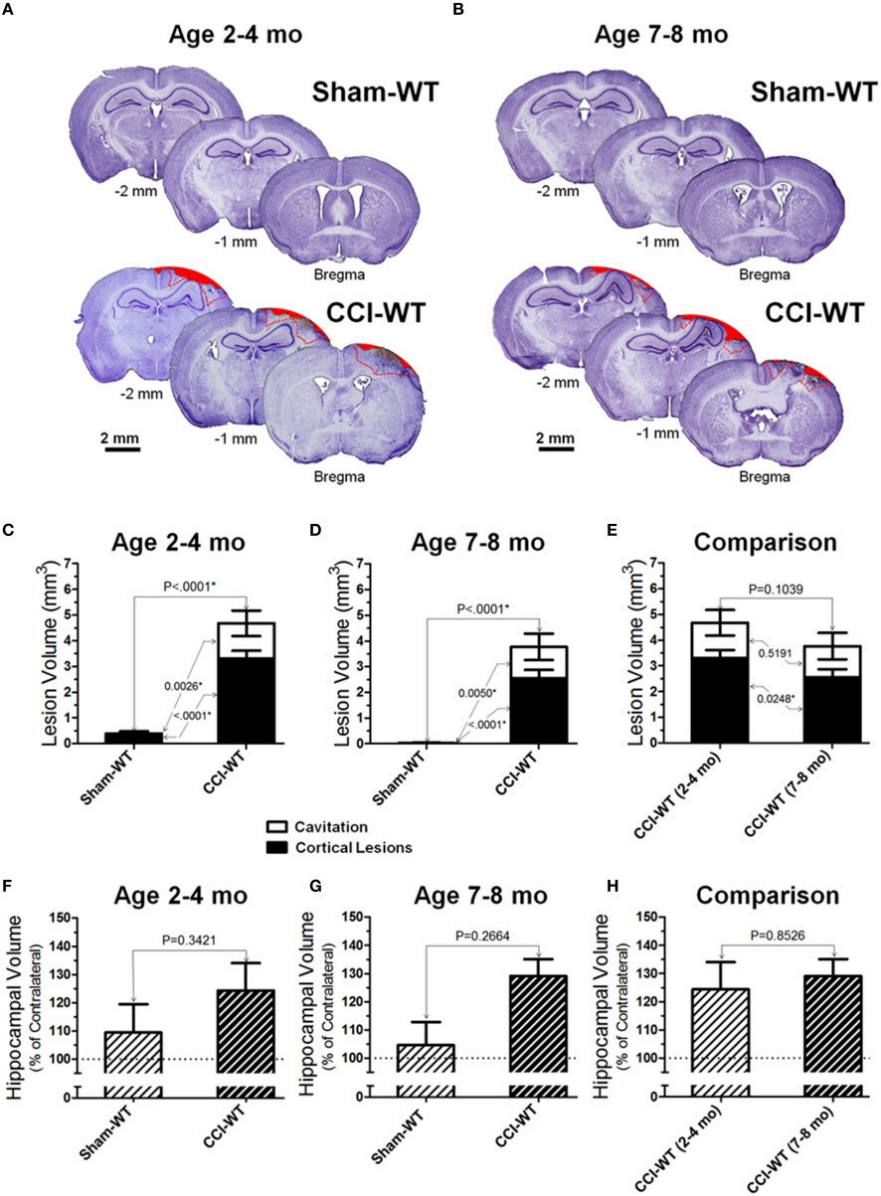
**Age-dependent effects of the experimental TBI on anatomical outcomes**. **(A**,**B)** The representative microphotographs of cresyl violet-stained brain sections obtained at 48 h after CCI and sham injury in adult and older adult WT mice, respectively. In each panel, three brain sections were cut from the same mouse within 1 mm apart posteriorly from the bregma (coordinates from 0 to −4 mm). The text and numbers denotes approximate distance from the bregma in posterior direction. Areas filled with red represent cavitation and red dotted lines represent boundaries of cortical lesions and cavitation areas used for quantitative histopathological analyses. **(C-E)** Comparison of lesion volumes between sham- and CCI-injured WT mice in adult and older adult age cohorts: **(C,D)** and between CCI-injured WT mice of different ages **(C)**, respectively. **(F-H)** Comparison of relative hippocampal volumes between sham- and CCI-injured WT mice in adult and older adult age cohorts **(C,D)**, and between CCI-injured WT mice of different ages **(E)**, respectively. The numbers shown on the graphs represents *P*-values from the multi-factor ANOVA an *post-hoc* statistical analyses performed using Student's *t*-test to compare values between matched groups (*n* = 5−10).

Figures 3A,B demonstrate representative microphotographs of brain sections obtained from WT sham and CCI-injured animals in the two age cohorts. At the 48 h time point in the adult and older adult age cohorts, CCI consistently produced significant anatomical pathologies in the cortex assessed by cresyl violet histo-stereological analysis, including cortical lesions and complete loss of brain tissue. Cortical lesions were characterized by morphological alternations, including characteristic changes in cellular morphology and cell density. These changes primarily reflect neurodegenerative processes, neuronal death, and the presence of diffuse parenchymal hemorrhages, which are representative of microvascular injury in cortical tissue surrounding the impacted area. In sham animals of both age cohorts, no (or only marginal) alterations were observed in the cortical tissue histology due to craniotomy surgery. Figures 3C,D represent analyses of distribution of contusion volume between volumes of lesioned cortical tissue and cavitation in CCI animals compared to the sham group. To determine possible changes in the patterns of brain lesions following experimental TBI, the injury volumes were analyzed by measuring and statistically comparing the volumes of cortical lesions and cavitation separately, and the data are presented as a contingency graph.

Cortical lesions were defined as histological alterations evident with cresyl violet staining, including cell loss (i.e., light stromal staining without or with markedly decreased nuclear staining), changes in cellular density and nuclear morphology (i.e., altered shape, shrinkage, and intensively stained and condensed nuclei), “penumbra” (i.e., the area adjusted to the injury core with evident signs on secondary injuries such as the aforementioned morphological changes), and small hemorrhages, whereas cavitation was defined as total loss of brain tissue or hematoma without visible cresyl violet staining. The measurements were made in brain segments between bregma coordinates from 0 to −2 mm, which covered all major brain segments including hippocampus and cortical regions associated with functional neurobehavioral outcomes. To compare the differences in cortical lesion, cavitation, and total contusion volumes between surgery groups (i.e., sham and CCI) and animal age, statistical analyses were performed using multi-factor ANOVA and *post-hoc* Student's test for pairwise comparison. The results demonstrate significant differences in all measures of cortical injury volume in CCI groups compared to sham-operated mice in both age cohorts (Figures 3C,E). There were significant effects of both factors (i.e., surgery type and age) on the cortical lesion and total contusion volumes (<0.0001 and *P* = 0.0003, respectively) and a significant effect of surgery factor on the cavitation volume measured (*P* = 0.0001). There was a statistically significant difference in cortical lesion volumes between CCI groups of the adult and older adult cohort (*P* = 0.0248), whereas there was no statistical difference between cavitation and total contusion volumes measured in these groups (Figure 3E). Interestingly, some marginal alterations in the brain that might be characterized as cortical lesions were observed in some animals from sham-surgery groups and there was a significant difference between the volumes of these lesions in sham groups of adult and older adult cohorts (*P* = 0.0017).

In most of the animals from the CCI groups in both age cohorts, morphological distortion of ipsilateral hippocampi and localized hippocampal edemas were noticeable on all brain sections where the hippocampus is present, with the most apparent presentation on the sections between about 1 and 2.5 mm posterior from the bregma. In some animals, hippocampal swelling was not obvious; ipsilateral hippocampus size was possibly reduced due to concurrent neuronal tissue degeneration (Glushakov et al., 2015). Two-way ANOVA and *post-hoc* comparison using Student's *t*-test revealed no significant changes in hippocampal volumes compared to sham in adult and older adult cohorts (Figures 3F,G). The analyses of hippocampal swelling has also demonstrated that the values of relative hippocampal volumes 48 h following CCI were not significantly different between WT mice from the adult and older adult cohorts (Figure 3H).

**Figure 4:**
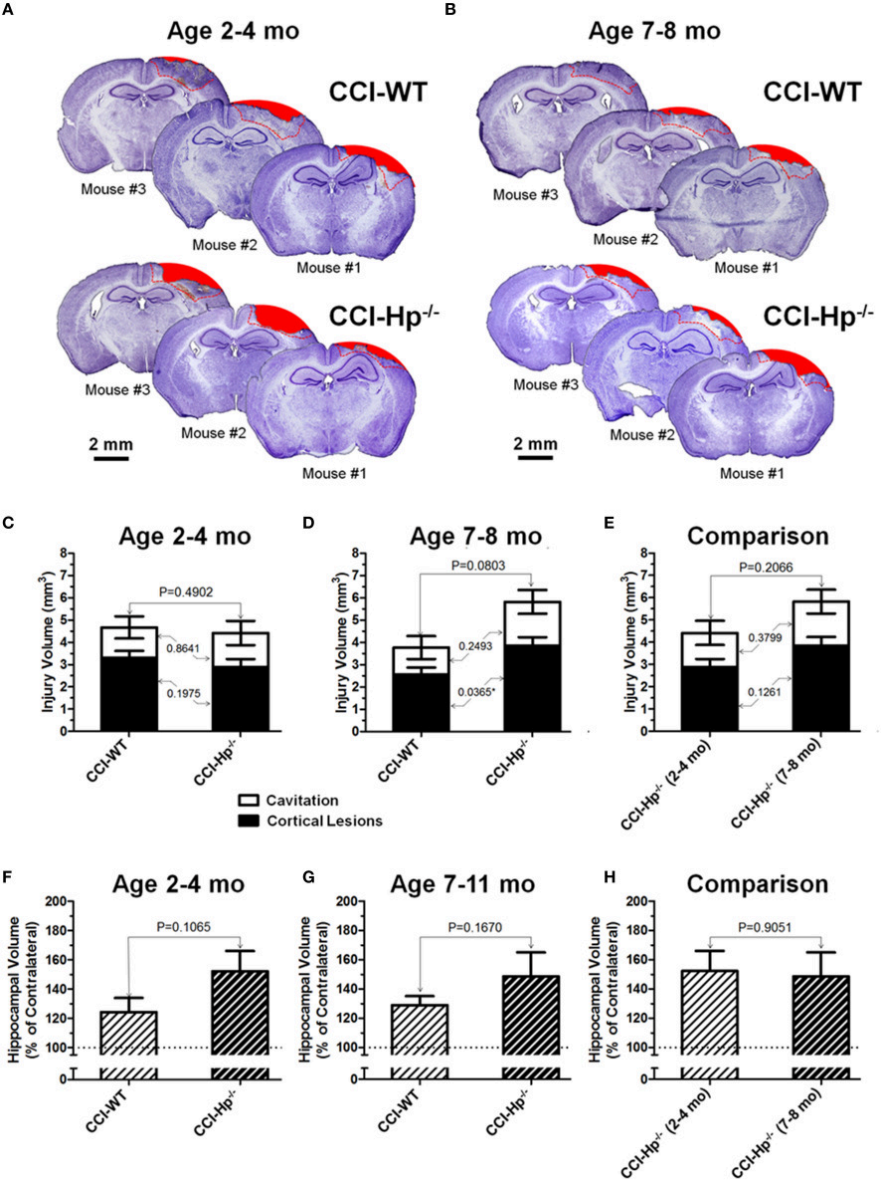
**Effects of the Hp knockout on anatomical outcomes following CCI in different age cohorts**. **(A,B)** The representative microphotographs of cresyl violet-stained brain sections obtained at 48 h after CCI in adult and older adult WT and Hp^−∕−^ mice, respectively. In each panel, three examples of the brain sections from three different animals from the same experimental group (marked as Mouse #1–3). The example brain section from each mouse was cut within coordinates from −1 to −2 mm. Areas filled with red represent cavitation and red dotted lines represent boundaries of cortical areas covered by cortical lesions used for quantitative histopathological analyses. **(C-E)** Comparison of the lesion volumes between CCI-injured WT and Hp^−∕−^ mice in the adult and older adult age cohorts **(C,D)**, and between the CCI-injured WT mice from different age cohorts **(E)**, respectively. **(F-H)** Comparison of relative hippocampal volumes between WT and Hp^−∕−^ CCI-injured mice in adult and older adult age cohorts **(C,D)**, and between CCI-injured Hp^−∕−^ mice of different ages **(E)**, respectively. The numbers shown on the graphs represents *P*-values from the multi-factor ANOVA an *post-hoc* statistical analyses performed using Student's *t*-test to compare values between matched groups (*n* = 4−10).

Figures 4A,B demonstrate examples of brain sections within the same bregma coordinates (from −1 mm to −2 mm) showing typical variability of anatomical brain pathologies between individual animals in WT and Hp^−∕−^ mice of adult and older adult cohorts. To compare the differences between anatomical outcome measures including cortical injury volume (i.e., cortical contusion, cortical lesions, and cavitation) and hippocampal swelling in WT and Hp^−∕−^ mice in two age cohorts, a multi-factor ANOVA and *post-hoc* pairwise comparison was performed using Student's test. The statistical analyses revealed the significant interaction of age and genotype in cortical lesion volume measures following CCI (*P* = 0.0172). The quantitative analyses revealed that there were no statistical differences between total contusion volumes of WT and Hp^−∕−^ mice in both age cohorts (Figures 4C,D) and between Hp^−∕−^ mice from adult and older adult cohorts (Figure 4E). Similarly, comparing hippocampal volumes in CCI-injured animals revealed no statistically significant differences between WT and Hp^−∕−^ mice in any age cohort or between Hp^−∕−^ mice from adult and older adult cohorts (Figures 4F–H). Interestingly, in the older adult age cohort, although total cortical lesion was not significantly different between WT and Hp^−∕−^ mice, the volume of cortical lesions in Hp^−∕−^ was significantly increased compared to WT mice (*P* = 0.0365). There was no significant difference observed between volumes of cavitation in these groups. Only marginal anatomical alteration was detected in sham Hp^−∕−^ mice in both adult and older adult cohorts (data not shown).

### Effects of the Hp knockout on astrocytic and microglial responses following CCI in the different age cohorts

**Figure 5:**
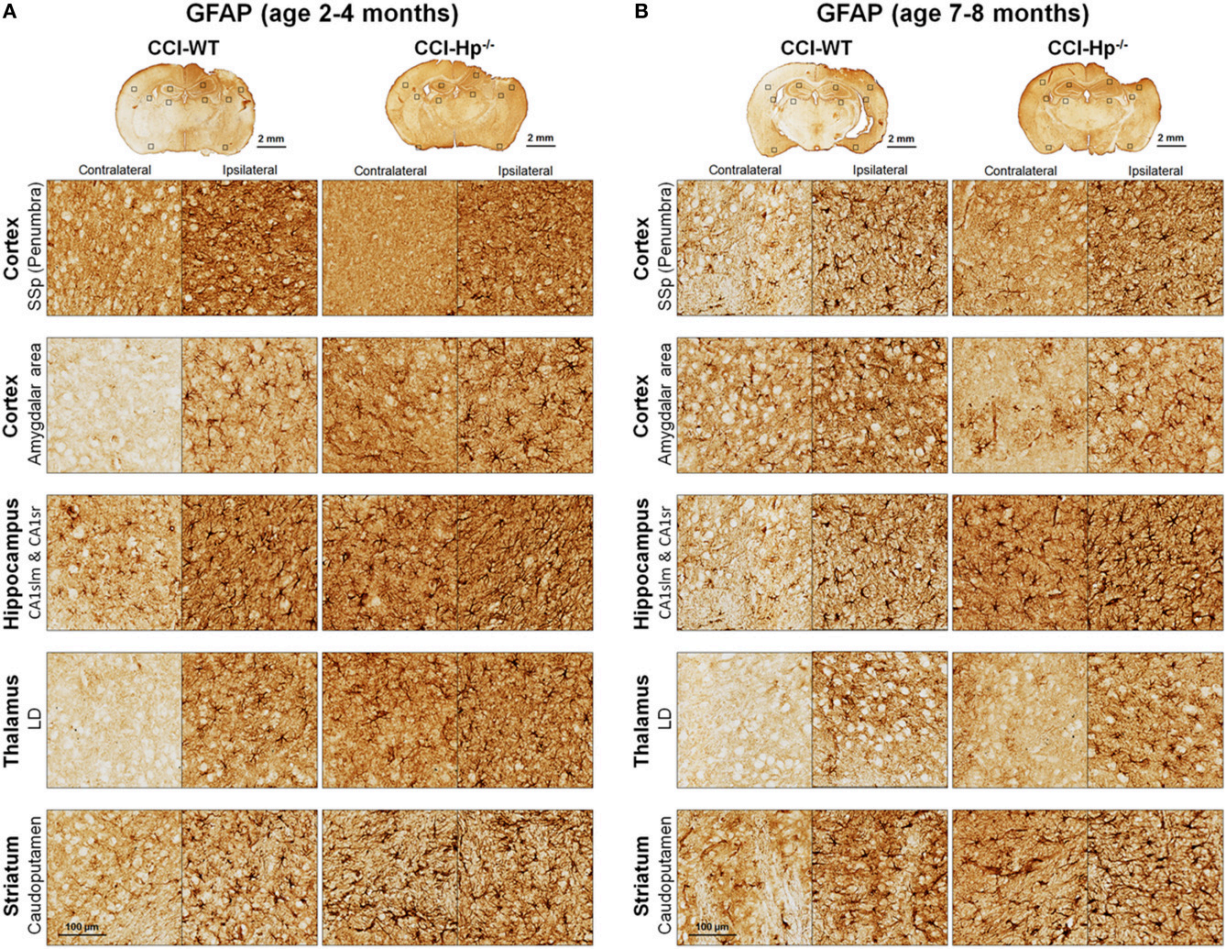
**Effects of Hp on the reactive astrocyte immunoreactivity at 48 h after CCI in 2–4 mo-old (adult) and 7–8 mo-old (older adult) mice**. **(A,B)** The representative microphotographs of GFAP immunostainings in the “penumbral” part of the primary somatosensory area (SSp) of cerebral cortex, posterior part of cortical amigdalar areas, lacunosum-moleculare (CA1slm), and stratum radiatum (CA1sr) fields of hippocampal CA1 area, lateral dorsal nucleus (LD) of thalamus. Identification of the brain structures and usage of the histological acronyms in this and other figures were achieved using Allen Mouse Brain Atlas (Lein et al., 2007).

**Figure 6:**
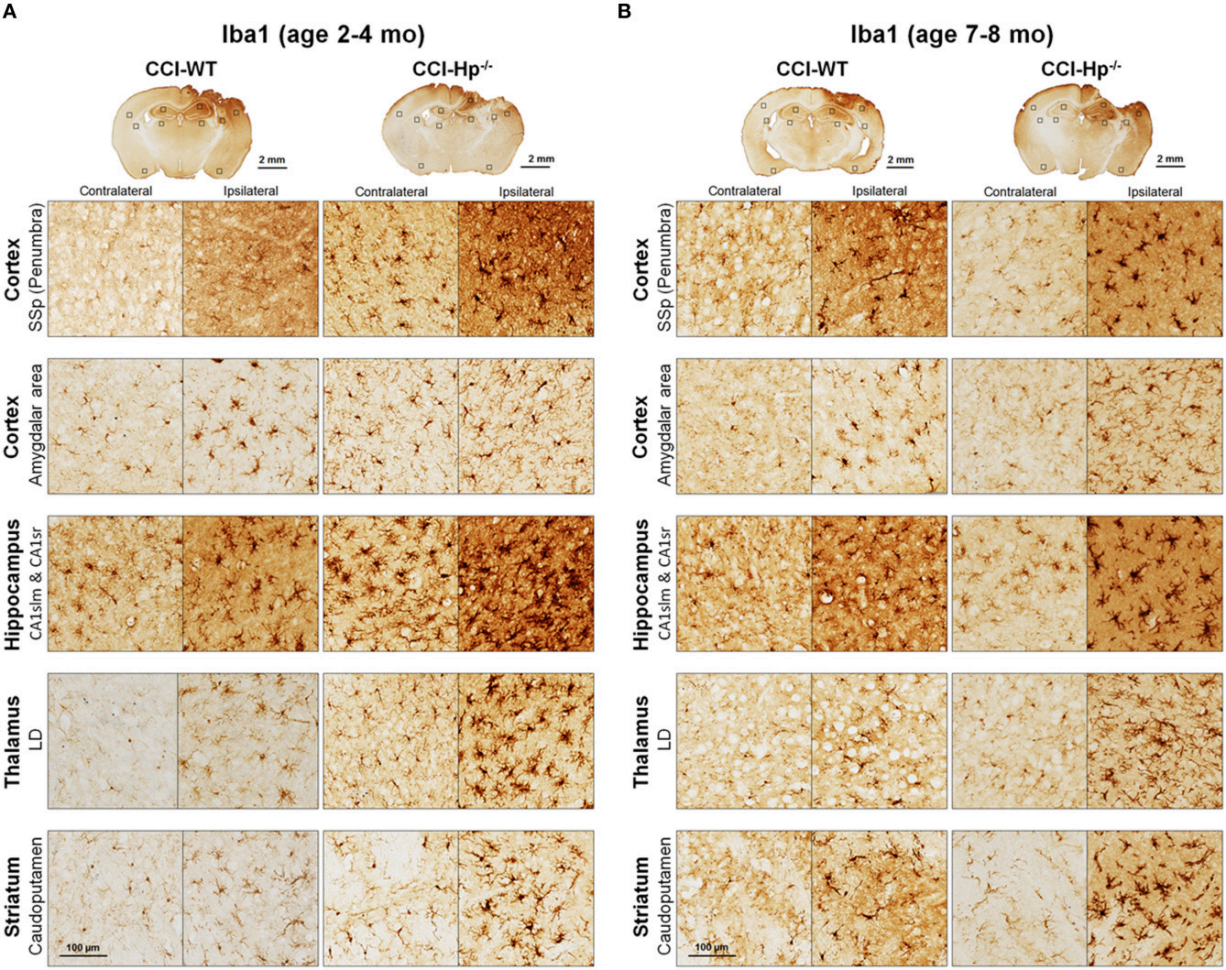
**Effects of Hp knockout on the activated microglia immunoreactivity at 48 h after CCI in 2–4 mo-old (adult) and 7–8 mo-old (older adult) mice**. **(A,B)** The representative microphotographs of Iba1 immunostained brain sections. The acronym definitions are the same as those in the legend of Figure 5.

**Figure 7:**
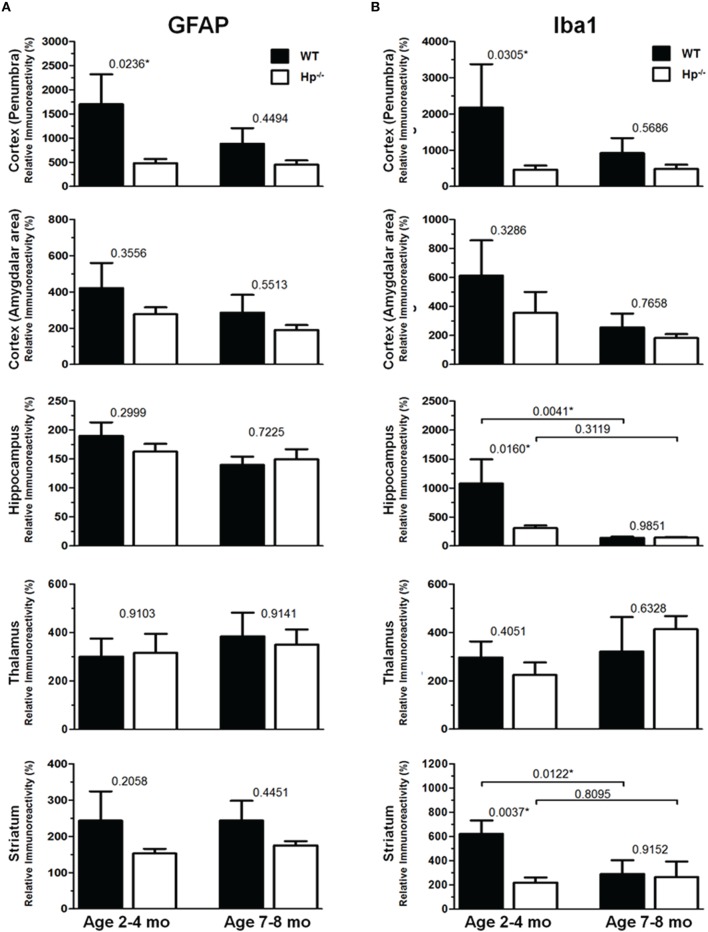
**Quantification of GFAP and Iba1 immunoreactivities at 48 h after CCI in in 2–4 mo-old (adult) and 7–8 mo-old (olde adult) mice**. The bar graphs show quantitative analyses of GFAP **(A)** and Iba1 **(B)** immunostained brain sections presented as relative immunoreactivity of in the selected ipsilateral brain regions (shown in the Figures 5, 6) normalized to the immunoreactivity values calculated in their contralateral counterparts. The numbers above bars in each graph represents *P*-values obtained by using multi-factor ANOVA and the *post-hoc* Student's *t*-test (*n* = 4–8). ^*^indicates significant difference.

Because the changes in glial responses to the CCI injury reflected in proliferation of glial cells and upregulation of specific markers are not definitely evident on the cresyl violet-stained sections, to study the prospective effects of Hp on the activation of reactive astrocytes and microglial cells, the GFAP and Iba1 immunostainings were performed on the brain sections obtained from WT and Hp^−∕−^ mice of two age cohorts, respectively. At 48 h after CCI, there was an apparent increase in immunoreactivity for both glial markers, glial cell proliferation, and changes in morphology of corresponding glial cells. Figures 5, 6 demonstrate representative microphotographs of GFAP- and Iba1-immunostained brain sections in two age cohorts, respectively, including zoomed selected areas of brain sections with the most extensive glial responses to the experimental TBI to demonstrate characteristic alteration in glial cell morphology. The morphological examination suggested some tendencies in the increased GFAP and Iba1 and in contralateral and ipsilateral brain regions of Hp^−∕−^ mice of both age cohorts. The findings of immunohistochemical experiments in adult WT mice are consistent with our previously published data showing significant increases in GFAP and Iba1 immunoreactivity in CCI compared to sham-injured animals. To assess the level of astrocytic and microglial activation, first, quantitative immunohistochemical analyses were performed separately in the major brain segments located within the CCI impact area with increments of 1 mm starting from the bregma (a total of four segments from 0 to −4 mm). However, the results revealed that there were no significant differences between relative immunoreactivities in 1-mm-thick segments within any brain region (multi-factor repeated measurement ANOVA with *post-hoc* Student's *t*-test; data not shown). Thus, we performed further analyses using the relative immunoreactivity values averaged for all four segments. Figure 7 demonstrates a summary of the results of quantification of immunohistochemical stainings shown in Figures 5, 6. The comparison of GFAP and Iba1 immunostainings between WT and Hp^−∕−^ mice in adult and older adult cohorts revealed that the results of statistical analyses are demonstrated in Figure 7 as *P*-values above bar graphs between corresponding groups and above horizontal bars in selected panels to show the significant difference between groups of the same genotype from different age cohorts (ANOVA with *post-hoc* Student's *t*-test).

## Discussion

This study examined for the first time the potential roles of the Hp pathway in TBI using two age cohorts of WT and Hp^−∕−^ mice. The results suggest that the role of Hp in TBI is multi-factorial and age dependent. Because Hp is involved in clearance of generally toxic free hemoglobin and has antioxidant properties (Campbell et al., 2005), its injury-induced upregulation would contribute to the neuroprotective compensatory mechanism (Vejda et al., 2002). Although there is no clear consensus, preclinical studies from Dr. J. Aronowski and his colleagues have suggested that Hp might be expressed in the brain and its induction would be sufficient to be protective in experimental intracerebral hemorrhage (Zhao et al., 2009, 2011). The results of our study suggest that global deletion of the Hp gene is associated with statistically significant better short-term functional outcomes following experimental TBI in 2–4 mo-old (adult) mice, whereas no such effects were observed between WT and Hp^−∕−^ 7–8 mo-old (older adult) mice, and there were no statistical differences between volumes of total contusion, cortical lesions, and tissue loss in WT and Hp^−∕−^ of both age cohorts. Statistical differences were also observed in the gliosis outcomes, i.e., astrogliosis and microgliosis.

Hp is involved in the acute phase response to systemic or local tissue injuries by cytokine-triggered hepatocytic synthesis and release into circulation of several defensive proteins (Wilcockson et al., 2002; Petersen et al., 2004; Campbell et al., 2005). Although limited preclinical studies provide evidence that Hp might be expressed in the brain (Zhao et al., 2009, 2011), it is more likely that increased CSF and brain levels of Hp observed after TBI might essentially result from blood-brain-barrier breakdown (Liu and Sturner, 1988; Bell et al., 1997). It has long been recognized that elevated serum Hp after TBI is produced mainly from the liver (Bowman and Kurosky, 1982; Hoj et al., 1984; Yang et al., 2013) it peaks approximately 24 h after injury and its levels are associated with increased cytokine levels (Bell et al., 1997; Amick et al., 2001). The latter observation is also supported by a previous immunohistochemical clinical study performed in the post-mortem brain of TBI patients that provided solid evidence that the spatiotemporal profiles of increased levels of Hp and several other plasma proteins are associated with blood-brain barrier breakdown and the extravasated plasma proteins and their subsequent uptake by the glial cells (Liu and Sturner, 1988). However, there is evidence of complex roles of Hp expression and its phenotypes in different types of brain injuries. Hp is a potent antioxidant and its increased expression has been documented in different brain injuries that are not directly associated with brain hemorrhages. Experimental studies have documented significant changes in Hp expression in the plasma of rats undergoing experimental transient focal cerebral ischemia (Chen et al., 2011). In a TBI model, Hp phenotypes were associated with differential neuropsychological outcomes; however, in contrast to clinical and preclinical data in subarachnoid brain hemorrhage, the “high” affinity Hp 1-1 phenotype was associated with worse outcomes (Anderson et al., 2009).

The neurological outcomes assessed using NDS and anatomical outcomes (i.e., brain lesions and cortical tissue) in adult WT mice observed in this study were consistent with our previously published data obtained in C57BL/6 mice within this age range (Glushakov et al., 2013, 2014, 2015). Not surprisingly, the NDS was increased in older adult mice of both WT and Hp^−∕−^ genotypes in the experiments with the same magnitude of the experimental TBI. In the sham group, WT mice from the 7–8 mo-old older adult cohort had a range of NDS that was seemingly higher compared to that observed in WT 2–4 mo-old adults from the sham group normally showing no detectable or only marginal neurological deficits, although there were no statistically significant differences between sham groups of WT mice from these two age cohorts. This observation suggests that some older adult animals have increased vulnerability to surgical procedures, including craniotomy performed in the sham groups. However, no such changes in the NDS variability were observed in the sham Hp^−∕−^ mice from both age cohorts and the ranges of NDS in these mice were within the range of 2–4 mo-old WT mice from the sham group.

Based on analyses of cresyl violet-stained sections, significant cortical and hippocampal pathologies were evident at the 48 h time point used in this study in all CCI-injured groups. The anatomical cortical injury was quantified as a cortical contusion volume, which was identified by characteristic tissue loss of brain tissue, neuronal death, and alteration in cellular morphology and integrity of brain structures and intracerebral hemorrhages. As we previously reported, in the CCI model, based on evident alterations in the hippocampus that are not directly affected by impact, the overall brain lesions could be categorized into two groups with prevalence reflecting severity of secondary injuries: type I and II, which are characterized by morphopathological changes in the ipsilateral hippocampus with remaining structural organization and by a complete loss of the ipsilateral hippocampus, respectively (Glushakov et al., 2015). The cortical cavitation is characteristic of both of these lesion types, although it is more prominent with later injury-onset time points (Glushakov et al., 2013).

It is well recognized that neuronal death and loss of brain tissue reflects secondary injury predominantly resulting from necrotic calpain-induced proteolysis (Wang et al., 2006; Saatman et al., 2010). In addition, our previous data have shown that the overall brain lesions might be categorized by the changes in the hippocampal morphology with seemingly abrupt transformation from hippocampal edema to hippocampal degeneration (lesion types I and II, respectively) resulting either from increasing severity of impact (e.g., increased compression distance) or from the deleterious effect of pharmacological intervention even with mild-to-moderate initial impact reflecting the severity of secondary injuries (Glushakov et al., 2015).

CCI parameters used in this study allowed one to induce brain injury by partial direct compression of the cortical tissue with rigid impactor, whereas the brain structures located underneath the cortical injury site are affected indirectly and with gradient decreasing pressure that likely would not cause meaningful mechanical damage. However, in this study, the prevalence of hippocampal alterations in most animals reflected type-I lesions, which are characterized by a marked increase in the ipsilateral hippocampal volume and distortion of hippocampal shape without substantial loss of hippocampal tissue, while in some animals, the ipsilateral hippocampi were of comparable size and shape or even had some reduction of ipsilateral hippocampal size, suggesting variability in ranges of responses to injury in individual animals.

Immunohistochemical experiments with GFAP and Iba1 stainings performed at the 48 h time point after experimental TBI revealed significant proliferation and changes in cell morphology of reactive astrocytes and activated microglial cells in selected brain regions consistent with our previous published data obtained with the same experimental parameters (Glushakov et al., 2013, 2014). The upregulation of these immunomarkers and proliferation of glial cells were observed in cortical lesioned tissue, including “penumbral” areas, located underneath the injury, and evident with histophatological examination using cresyl violet staining; and also, to a lesser extent, in the brain regions not directly affected by the CCI impact without evident histopathological changes (cresyl violet) throughout the whole ipsilateral cerebral cortex even with seemingly similar immunoreactivity intensity and cellular morphology up to distal cortical amigdalar areas; and ipsilateral brain structures that are not directly impacted, including the hippocampus, and some areas of the thalamus and striatum. The results of these experiments indicate that there were differences in microglial and astrocytic reactivity between Hp^−∕−^ and WT in selected brain regions of the adult but not older adult age cohort, suggesting that Hp might be involved in glial or inflammatory responses, or affecting secondary injuries to the levels that are reflected in changes in upregulation of astrocytic and microglial markers or proliferation of glial cells. There were also some apparent tendencies in the upregulation of glial cells in selected contralateral regions of Hp^−∕−^ mice of both age cohorts that might result from presumed impairment of overall defensive mechanisms due to deletion of Hp; establishing their potential clinical importance in brain injuries requires more detailed investigation. Although reactive astrocytes have been implicated in playing a role in the uptake of Hp and other extravasated plasma proteins (Liu and Sturner, 1988) the result of GFAP, as well as Iba1, IHC experiments in Hp^−∕−^ did not support the astrocytic or microglial involvement in clearance of assumed Hp-hemoglobin complexes following TBI. The roles of astrocytes microglia in brain injury are complex, including both beneficial and detrimental effects (Loane and Byrnes, 2010). On the other hand, it is also recognized that increased upregulation of astrocytic and microglial markers is associated with the extent of anatomical and behavioral deficits. In our study, the significantly decreased upregulation of GFAP and Iba1 in selected brain regions of Hp^−∕−^ mice may indicate involvement of Hp in these inflammatory responses. The effect of aging in experimental models of TBI is well recognized, including neurological outcomes, neuronal brain pathology, and blood-brain barrier function (Onyszchuk et al., 2008; Sandhir et al., 2008; Lee et al., 2012; Timaru-Kast et al., 2012). However, little is known about the effects of Hp expression on brain injuries, and in our study, we have made an attempt to uncover possible dependencies of Hp roles with age using WT and Hp^−∕−^ mice. The results of this study revealed significant associations of both age and genotype factors with several neurobehavioral, anatomical, and immunohistochemical outcome measures. Interestingly, the data suggest that improved NDS in Hp^−∕−^ might be associated with decreased upregulation of both GFAP and Iba1 in the cortical areas located adjacent to the injury site and Iba1 upregulation in selected hippocampal and striatal regions in adult CCI-injured mice. However, these effects of Hp deletion were lost with aging.

In terms of some limitations of this study, although we believe that using of Hp^−∕−^ mice is one of the currently reasonable available approaches to test our hypotheses, the use of genetically modified mice, in general, has inherent limitations due to activation of possible compensatory mechanisms to restore the organism's functions. In particular, it might be critical in case of “protective” proteins such as Hp that might potentially affect the physiological state and affect responsiveness to the experimental injury. Thus, some differences in the outcomes in adult and older adult mice might be attributed to the chronic ablation of a component of the acute phase response system. In addition, the translational potential of this study might be limited because of the heterogeneity of human Hp phenotypes in contrast to the homogeneity of Hp in mice and other animals, and thus using animal models may not reflect the whole spectrum of the various Hp-involving pathways. Another limitation of the study is using only an acute time point with the given experimental conditions reflecting only the Hp roles in short-term outcomes, whereas some results of our study suggest that long-term outcomes might be different.

In conclusion, this study provides an insight into the prospective roles of Hp in TBI and other acute brain injuries, especially those with complex mechanisms. The data of this study suggest that systemic Hp might interact with the intrinsic brain's mechanisms of hemoglobin clearance and/or antioxidative protective pathways affecting short-term clinical outcomes. The data also suggest that ablation of Hp might affect the organism's responsiveness to brain injuries and this effect is more prominent with aging. In addition, these results might be implicated in understanding the inconsistency in outcomes of clinical studies regarding the importance of Hp phenotypes in brain injuries. However, the potential roles of Hp in long-term outcomes warrant additional studies.

## Author contributions

AG designed, performed, and analyzed all experiments, wrote the manuscript, and trained RA in performing behavioral and histochemical experiments and data analyses; RA performed behavioral and histochemical experiments and analyses, and edited the manuscript; ET developed and provided the breeding stock of Hp^−∕−^ mice, and revised the manuscript; SD designed the experiments, assisted in the analyses, provided funding and expertise, and contributed to writing and revising the manuscript. All authors have accepted the final version of the manuscript.

## Funding

This work was supported by a grant from the McKnight Brain Research Foundation, Brain and Spinal Cord Injury Research Trust Fund (SD) and grants from the National Institutes of Health NS046400 and R01AT007429 (SD). The funders had no role in study design, data collection and analyses, decision to publish, or preparation of the manuscript.

### Conflict of interest statement

The authors declare that the research was conducted in the absence of any commercial or financial relationships that could be construed as a potential conflict of interest.
